# ECM remodeling features in reparative chondrocytes during knee osteoarthritis

**DOI:** 10.3389/fendo.2026.1773139

**Published:** 2026-04-13

**Authors:** Fei Li, Zhenfeng Zhang, Jiafeng Peng, Hongxing Zhang, Yingzong Xiong, Zhiwen Zheng, Ran Xu, Jiahao Ruan, Qiqi Yang, Zhenggang Lu, Xingfu Ma, Chao Wang, Bin Dai, Junchen Zhu

**Affiliations:** 1 The Second Affiliated Hospital of Anhui University of Traditional Chinese Medicine, Hefei, China; 2 Graduate School, Anhui University of Traditional Chinese Medicine, Hefei, China; 3 Changsha Medical University, Changsha, China; 4 Department of Spine Surgery, Anhui Provincial Hospital, Hefei, China

**Keywords:** extracellular matrix remodeling, knee osteoarthritis, pseudotime trajectory, reparative chondrocytes, single-cell RNA sequencing

## Abstract

**Background:**

Knee osteoarthritis (KOA) is characterized by progressive cartilage degeneration and disruption of extracellular matrix (ECM) homeostasis. Chondrocytes are not a homogeneous or static population during disease progression but exhibit pronounced functional heterogeneity. Although single-cell transcriptomic studies have identified multiple chondrocyte states in osteoarthritic cartilage, how these states dynamically relate to ECM remodeling and disease progression remains incompletely understood.

**Methods:**

We integrated multiple publicly available single-cell RNA sequencing datasets of human knee cartilage to construct a unified cellular atlas and systematically compared chondrocyte states between control and KOA samples. Differential expression analysis, functional enrichment, pseudotime trajectory inference, and cell–cell communication analysis were applied to characterize ECM-related chondrocyte states and their dynamic transitions. Key signaling cues identified from single-cell analyses were further evaluated using *in vitro* cultured human articular chondrocytes.

**Results:**

We observed a marked expansion of the previously described reparative chondrocyte population (RepC) in KOA cartilage. Rather than reflecting a simple increase in cell proportion, KOA-associated RepC exhibited enhanced ECM remodeling programs characterized by collagen reorganization and strengthened ECM–cell interactions. Pseudotime analysis positioned RepC downstream of proliferation chondrocytes and near a major branching region toward either regulator chondrocytes with further extension toward fibrocartilage chondrocytes or effector chondrocytes. In KOA, RepC was preferentially represented at mid-to-late pseudotime stages within the reconstructed trajectory framework. Cell–cell communication analysis suggested that RepC showed prominent inferred ECM-related interactions, particularly involving collagen and FN1–integrin pathways. Consistently, FN1 or TGF-β1 stimulation *in vitro* induced expression of multiple RepC-associated genes and enhanced SMAD2/3 phosphorylation, recapitulating key features of the RepC state observed in single-cell analyses.

**Conclusion:**

These findings highlight ECM remodeling features of reparative chondrocytes during KOA and support a state-centric view in which disproportionate representation of reparative states within the pseudotime trajectory framework is associated with maladaptive ECM remodeling in KOA.

## 1 Introduction

Knee osteoarthritis (KOA) is one of the most prevalent degenerative joint diseases in adults, with its pathological progression primarily characterized by progressive cartilage degeneration accompanied by disruption of extracellular matrix (ECM) homeostasis (1). Recent epidemiological analyses indicate that the global burden of KOA continues to rise, imposing an increasing strain on population health and healthcare systems worldwide (2). Against this backdrop, a deeper understanding of the interplay between chondrocyte functional states and dynamic ECM remodeling is essential for elucidating the pathogenic mechanisms of KOA. Accumulating evidence suggests that chondrocytes undergo dynamic transitions across multiple functional states during disease progression, encompassing proliferative, reparative, regulatory, effector, and fibrocartilage-associated programs (3, 4). Advances in single-cell transcriptomics have further revealed multiple chondrocyte subpopulations with distinct transcriptional profiles in osteoarthritic cartilage and have delineated lineage trajectories that progressively transition from homeostatic cells toward regulatory, effector, or fibrocartilage-associated states (5). Despite these advances in characterizing chondrocyte cellular heterogeneity, how such state transitions are mechanistically linked to dynamic ECM remodeling has not yet been systematically integrated.

The advancement of single-cell transcriptomic technologies has enabled the dissection of cartilage cellular heterogeneity and state transitions at single-cell resolution and, when integrated with trajectory inference and cell–cell communication analyses, has facilitated the identification of potential regulatory mechanisms underlying these processes (5–7). Among the identified chondrocyte subpopulations, reparative chondrocytes (RepC) have been reported in prior single-cell studies of osteoarthritic cartilage (8). However, these studies were primarily conducted on individual datasets and focused on other disease-associated populations, leaving the functional features, trajectory positioning, and intercellular communication patterns of RepC in KOA insufficiently characterized. To address these questions, this study integrates three independent publicly available human knee cartilage single-cell RNA sequencing datasets, comprising 193,409 high-quality cells, and applies a unified analytical framework to systematically compare cellular composition, state transitions, and ECM remodeling–related gene expression patterns between healthy and KOA cartilage. Furthermore, cell–cell communication analyses in combination with *in vitro* experiments were performed to explore the signaling regulatory pathways associated with these distinct cellular states. Collectively, this study aims to characterize ECM remodeling–related cellular states in KOA and to identify their potential regulatory determinants, thereby providing cell-scale evidence for understanding cartilage pathological remodeling.

## 2 Methods

### 2.1 Single-cell data acquisition and quality control

Three human knee cartilage single-cell RNA sequencing datasets (GSE169454, GSE220243, and GSE255460) were retrieved from the Gene Expression Omnibus (GEO) database (9). Raw expression matrices generated using the 10x Genomics platform were preprocessed using Seurat (v5.0). Standard quality control procedures were applied to exclude cells with fewer than 400 or more than 6,000 detected genes, fewer than 800 or more than 40,000 UMI counts, mitochondrial gene proportions exceeding 15%, ribosomal gene proportions exceeding 40%, or hemoglobin gene proportions exceeding 1% (10). Potential doublets were subsequently identified for each sample using DoubletFinder, and suspicious doublets were removed based on optimal parameter settings obtained from parameter sweeps (11). Following quality control, samples with markedly low cell numbers or failing to meet quality criteria were excluded. A total of 27 cartilage samples were ultimately retained for downstream data integration and analysis, including 9 control samples and 18 KOA samples.

### 2.2 Data integration, batch correction, and primary cell type annotation

Following quality control filtering, cells were processed using Seurat for normalization, highly variable gene selection, and data scaling. Technical batch effects arising from different samples and dataset sources were then corrected using Harmony, after which dimensionality reduction and clustering were performed in the integrated low-dimensional space. Major cell types were annotated based on canonical marker genes and used to construct a primary cell atlas of knee cartilage tissue.

### 2.3 Chondrocyte subpopulation identification

Based on the expression of canonical chondrocyte marker genes identified during primary annotation, all chondrocytes were extracted and constructed as an independent object. This chondrocyte subset was processed using the same analytical pipeline as described above, including normalization, highly variable gene selection, and Harmony-based batch correction, followed by clustering analysis in the integrated low-dimensional space (12). Distinct chondrocyte subpopulations were annotated according to their representative expression patterns in conjunction with previously reported chondrocyte classification frameworks to delineate intra-chondrocyte cellular heterogeneity (8) (**Table 1**).

**Table 1:** Representative marker genes defining six major chondrocyte subtypes.

Population	Marker genes
ProC (Proliferation chondrocytes)	C11orf96, BMP2, HMGA1
EC (Effector chondrocytes)	CHRDL2, FRZB, CYTL1
RegC (Regulator chondrocytes)	CHI3L1, CHI3L2
RepC (Reparative chondrocytes)	CILP2, CILP, OGN
HomC (Homeostasis chondrocytes)	HSPA1B, HSPA1A, HSPA6, DDIT3, JUN
FC (Fibrocartilage chondrocytes)	MMP2, COL1A1, COL1A2

### 2.4 Differential expression analysis and functional enrichment analysis

Differential expression analysis was conducted using expression matrices without batch correction. Genes enriched in specific chondrocyte subpopulations relative to all remaining chondrocytes were identified using the FindMarkers function in Seurat with Benjamini–Hochberg correction (13). For characterization of RepC under KOA conditions, differential expression analysis was performed within the KOA group by comparing RepC with the remaining chondrocyte subpopulations. Significantly upregulated genes were then subjected to Gene Ontology (GO) biological process and Kyoto Encyclopedia of Genes and Genomes (KEGG) pathway enrichment analyses to characterize the associated functional features (14, 15). In addition, for transparency and robustness assessment, an auxiliary comparison between KOA and control RepC was performed at the cell level and further examined using a sample-level pseudobulk strategy, and the concordant results are summarized in **Supplementary Table 2**.

### 2.5 Pseudotime trajectory analysis of chondrocytes

Based on Harmony-corrected dimensionality reduction results of chondrocytes, Slingshot was employed to construct trajectories of cell state transitions and to estimate global and branch-specific pseudotime for the subset of chondrocyte states included in the trajectory framework. Pseudotime values were subsequently normalized, and dynamic changes in gene expression and functional module scores along the pseudotime axis were assessed to characterize chondrocyte features along the inferred trajectory framework (16). In addition, pseudotime was partitioned into multiple intervals to quantify the compositional distribution of different subpopulations across the reconstructed trajectory, and relative proportions of control and KOA cells along the pseudotime trajectory were compared to capture changes in chondrocyte state distribution associated with the inferred disease-related trajectory structure.

### 2.6 Cell–cell communication analysis

Based on the chondrocyte expression matrix, CellChat was employed to construct intercellular communication networks separately for the control and KOA groups (7). Sender and receiver populations were defined for each chondrocyte subpopulation, and potential communication relationships were inferred using the built-in ligand–receptor database. Interaction probabilities, pathway-level communication strength, and network centrality metrics were subsequently calculated. The communication networks from the two groups were then integrated to compare global communication patterns and to delineate changes in inferred incoming and outgoing communication activities of specific chondrocyte subpopulations between control and KOA conditions.

### 2.7 Cell culture

Human articular chondrocytes (HCCs) were purchased from BluefCell (Shanghai BluefCell Life Science Co., Ltd., China; product number BFN60810515). Upon receipt, cells were thawed and cultured according to the manufacturer’s instructions. All experiments were conducted using early-passage cells (passage 2, P2) to ensure cellular stability. Cells were maintained in medium composed of 90% DMEM, 10% fetal bovine serum (FBS), and 1% penicillin–streptomycin and incubated at 37 °C in a humidified atmosphere with 5% CO_2_. Experimental treatments were initiated when cell confluence reached approximately 80%. The experimental groups included a control group, a TGF-β1 (5 ng/mL) group, an FN1 (10 μg/mL) group, and a combined TGF-β1 plus FN1 treatment group. Following treatment, cells were collected for RNA and protein extraction.

### 2.8 Quantitative real-time PCR analysis

Total RNA was extracted from treated human knee articular chondrocytes using TRIzol reagent (Thermo Fisher Scientific; Cat. No. 15596026) according to the manufacturer’s instructions. RNA concentration and purity were determined using an ultramicro spectrophotometer, and only samples with A260/A280 ratios between 1.8 and 2.1 were used for subsequent analysis. One microgram of total RNA was then reverse-transcribed into cDNA using the HiScript II Reverse Transcription Kit (Vazyme, China; Cat. No. R223-01). Quantitative real-time PCR was performed with AceQ SYBR Green Master Mix (Vazyme, China; Cat. No. Q111-02) on a LightCycler 480 II system (Roche, Switzerland). The amplification protocol consisted of an initial denaturation at 95 °C for 5 min, followed by 40 cycles of denaturation at 95 °C for 10 s and annealing/extension at 60 °C for 30 s, with a melting curve analysis conducted at the end to confirm amplification specificity. Relative gene expression levels were calculated using the 2^−ΔΔCt method, with β-actin serving as the internal control. Transcript levels of CILP, OGN, MMP13, and FRZB were quantified according to the experimental design, and all primer sequences are provided in **Table 2**.

**Table 2:** Primer sequences used for quantitative real-time PCR.

Primer name	Primer sequence (5’-3’)
CILP	F-TCCCAAGGCATCTAAGCGTC
	R-AGCATCGTCTGTCTCCCTGA
OGN	F-CCATCATTACCAACCAAGAAAGA
	R-GGTGGTACAGCATCAATGTCA
MMP13	F-AAATTATGGAGGAGATGCCCATT
	R-TCCTTGGAGTGGTCAAGACCTAA
FRZB	F-ATTTTCCTATGGATTCAAGTACTG
	R-TTGACTTTCTTACCAAGCCGATCCTT
β-Actin	F-AGAAAATCTGGCACCACACC
	R-CCATCTCTTGCTCGAAGTCC

### 2.9 Western blot analysis

Cellular proteins were extracted using RIPA lysis buffer supplemented with PMSF and phosphatase inhibitors on ice. Following centrifugation at 12,000 × g for 15 min, supernatants were collected, and protein concentrations were measured using a BCA assay kit. Equal amounts of protein (20–30 μg) were separated by SDS-PAGE and transferred onto 0.45 μm PVDF membranes. Membranes were blocked with 5% BSA at room temperature for 1 h and subsequently incubated overnight at 4 °C with primary antibodies. The primary antibodies included CILP (PA5-18553, Thermo Fisher Scientific; 1:1500), MMP13 (18165-1-AP, Proteintech; 1:1000), phospho-SMAD2 (ET1612-32, HUABIO; 1:5000), phospho-SMAD3 (ET1609-41, HUABIO; 1:5000), SMAD2 (12570-1-AP, Proteintech; 1:2000), SMAD3 (66516-1-Ig, Proteintech; 1:2000), and β-actin (66009-1-Ig, Proteintech; 1:20000). After washing with TBST, membranes were incubated with HRP-conjugated secondary antibodies (1:5000) at room temperature for 1–2 h. Protein bands were detected using an enhanced chemiluminescence (ECL) system and imaged with a FluorChem R imaging system. Band intensities were quantified using ImageJ software (v1.53) and normalized to β-actin. All experiments were independently repeated at least three times.

### 2.10 Statistical analysis

Statistical analyses were conducted using GraphPad Prism software (version 9.0). Quantitative data from *in vitro* experiments are presented as mean ± SD from three independent biological replicates. For *in vitro* experiments involving multiple treatment groups, differences among groups were evaluated using one-way ANOVA followed by Tukey’s *post hoc* multiple-comparison test. All statistical tests were two-sided, and a p value < 0.05 was considered statistically significant.

## 3 Results

### 3.1 Multi-dataset integration and identification of major cell types

**Figure 1:**
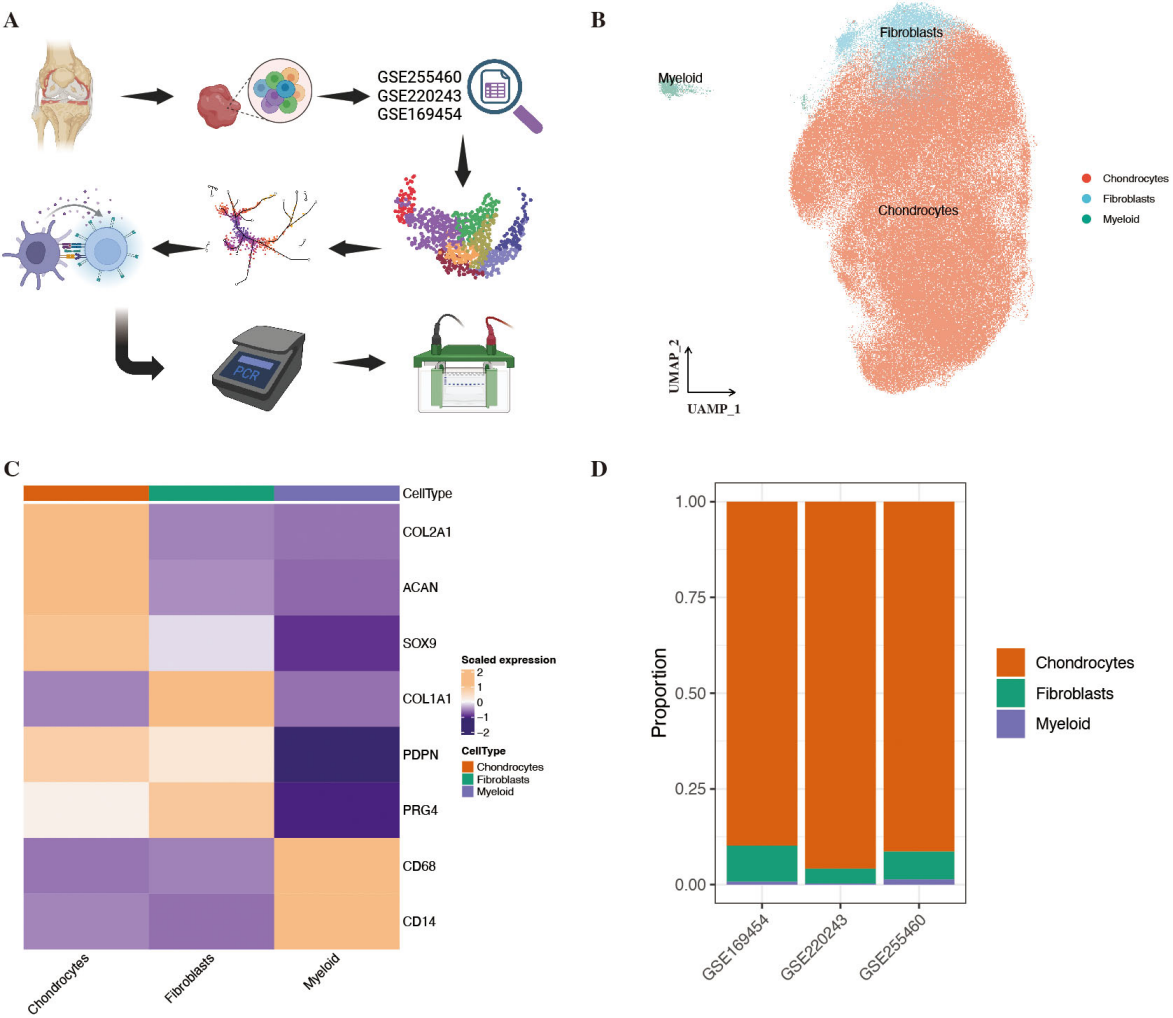
Integrated single-cell transcriptomic landscape of human knee articular cartilage. **(A)** Schematic overview of the study design. Single-cell RNA sequencing datasets from three independent cohorts (GSE169454, GSE220243, and GSE255460) were integrated to construct a unified cartilage cell atlas, followed by trajectory inference, cell–cell communication analysis, and experimental validation. **(B)** UMAP visualization of all cells after Harmony-based batch correction, showing major cell populations including chondrocytes, fibroblasts, and myeloid cells. **(C)** Heatmap displaying scaled expression of canonical marker genes used for major cell-type annotation, confirming clear separation among chondrocytes (COL2A1, ACAN, SOX9), fibroblasts (COL1A1, PDPN, PRG4), and myeloid cells (CD68, CD14). **(D)** Proportional composition of major cell types across individual datasets, demonstrating consistent enrichment of chondrocytes and effective integration across cohorts.

To construct a unified atlas of human knee cartilage cells, three publicly available single-cell RNA sequencing datasets (GSE169454, GSE220243, and GSE255460) were integrated (**Figure 1A**). After stringent quality control (**Supplementary Figure 1A**), a total of 193,409 high-quality cells were retained for downstream analyses, comprising 133,887 cells from 18 KOA samples and 59,522 cells from 9 control (CON) samples. Across the three datasets, GSE169454 contributed 48,576 cells, GSE220243 contributed 70,205 cells, and GSE255460 contributed 74,628 cells. Detailed per-sample cell counts are provided in **Supplementary Table 1**. Following Harmony-based batch correction, cells from different samples showed robust mixing in the low-dimensional embedding (**Supplementary Figure 1B**). Moreover, cells derived from the three datasets exhibited highly overlapping distributions (**Supplementary Figure 1C**), indicating effective removal of batch effects. Unsupervised clustering resolved three major cellular lineages, including chondrocytes, fibroblasts, and a minor population of myeloid cells (**Figure 1B**). Canonical lineage marker genes further confirmed cell identities: COL2A1, ACAN, and SOX9 were highly expressed in chondrocytes, whereas COL1A1, PDPN, and PRG4, together with CD14 and CD68, marked fibroblasts and myeloid cells, respectively (**Figure 1C**). The relative proportions of these three cell types were comparable across datasets, supporting the stability of cellular composition following data integration (**Figure 1D**).

### 3.2 Chondrocyte subpopulation identification reveals marked expansion of RepC in KOA

**Figure 2:**
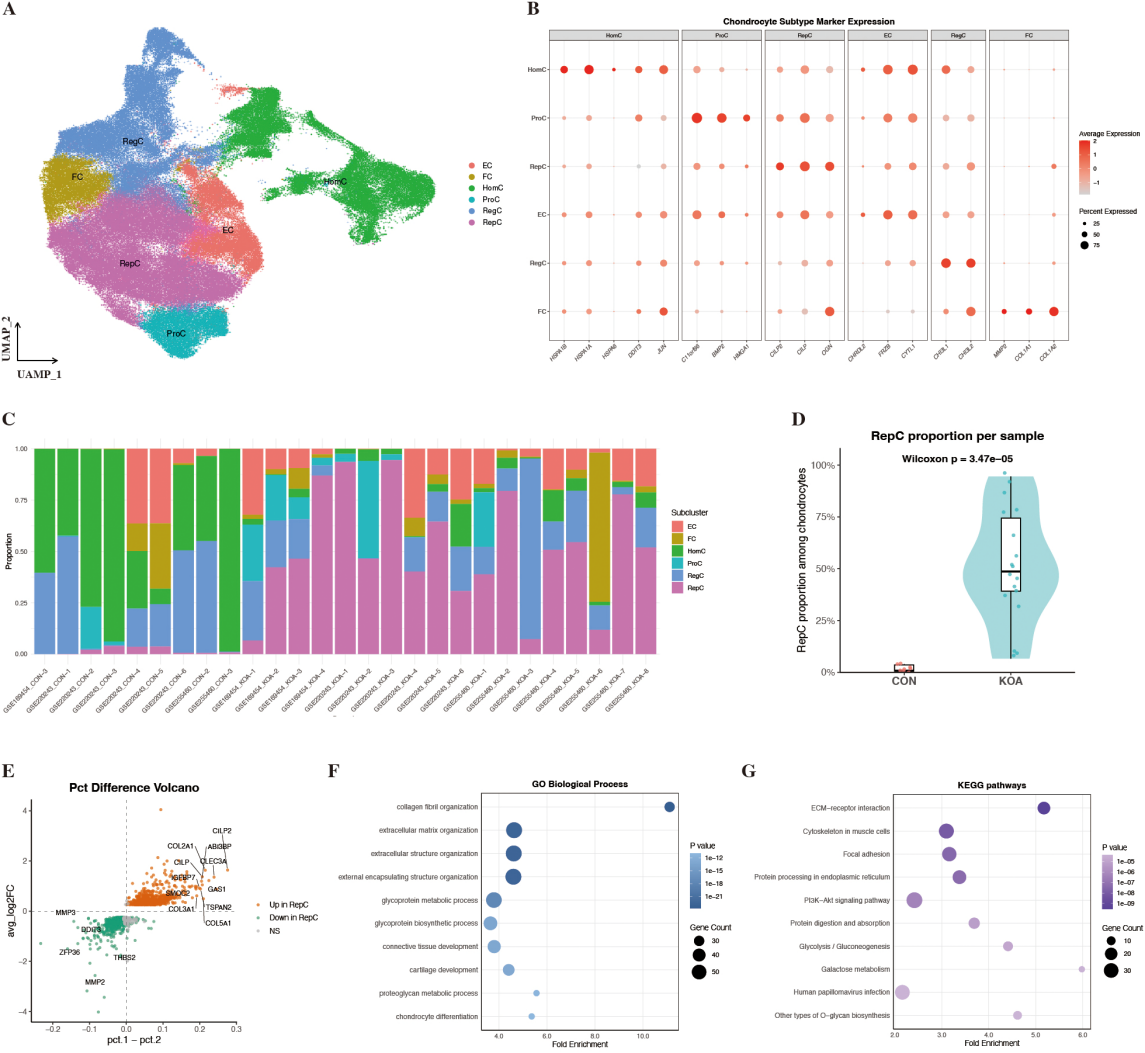
Identification of chondrocyte subpopulations and expansion of RepC in KOA. **(A)** UMAP projection of chondrocytes colored by annotated subtypes, including ProC, RepC, RegC, EC, FC, and HomC. **(B)** Dot plot showing the expression of representative marker genes for each chondrocyte subtype, supporting robust subtype annotation. **(C)** Stacked bar plots illustrating the relative proportions of chondrocyte subtypes across individual samples. **(D)** Quantification of RepC proportions among chondrocytes in control (CON) and KOA samples, revealing a significant expansion of RepC in KOA cartilage (Wilcoxon test). **(E)** Volcano plot of differential expression analysis highlighting genes enriched in RepC relative to other chondrocyte subpopulations within the KOA context. **(F, G)** GO and KEGG enrichment analyses of genes upregulated in RepC relative to other chondrocyte subpopulations within KOA.

Following extraction of chondrocytes from the integrated dataset, dimensionality reduction, clustering, and subpopulation annotation were re-performed to resolve intra-chondrocyte heterogeneity. Six chondrocyte subpopulations with distinct molecular signatures were identified, including HomC, ProC, RepC, RegC, EC, and FC (**Table 1**). In the UMAP low-dimensional embedding, these six subpopulations formed well-separated clusters, indicating robust and stable subpopulation delineation (**Figure 2A**). Analysis of representative marker gene expression supported the annotation of each subpopulation, with expression patterns generally consistent with previously reported chondrocyte functional states (**Figure 2B**). Comparative analyses demonstrated a pronounced shift in chondrocyte subpopulation composition in KOA cartilage, characterized by a significant increase in the relative abundance of RepC in KOA samples (**Figure 2C**). This enrichment of RepC was consistently observed across individual samples (**Figure 2D**). HomC showed an opposite distribution pattern and was predominantly represented in control samples (**Supplementary Figure 2**). Collectively, these results identify RepC expansion, together with relative depletion of HomC, as a major compositional feature of KOA cartilage.

### 3.3 RepC in KOA exhibits enhanced ECM remodeling–related programs

To further characterize the transcriptional features of RepC within the KOA context, differential expression analysis was performed within the KOA group, comparing RepC with the remaining chondrocyte subpopulations using Seurat’s FindMarkers function (Wilcoxon rank-sum test with Benjamini–Hochberg correction). In addition, for transparency, an auxiliary comparison between KOA and control RepC was performed at the cell level and further examined using a sample-level pseudobulk strategy, and concordantly upregulated genes are summarized in **Supplementary Table 2**. Genes upregulated in RepC within the KOA context were predominantly enriched in ECM structural proteins and fibrotic collagen–related components, including the representative ECM remodeling–associated factor CILP as well as collagen-related genes COL1A1, COL1A2, and COL3A1 (**Figure 2E**). Gene Ontology (GO) and Kyoto Encyclopedia of Genes and Genomes (KEGG) enrichment analyses further demonstrated that these upregulated genes were significantly enriched in pathways related to collagen fibril organization, extracellular matrix structural constituents, and ECM–receptor interaction (**Figures 2F, G**).

### 3.4 Pseudotime analysis positions RepC near a major branching region during chondrocyte state transitions

**Figure 3:**
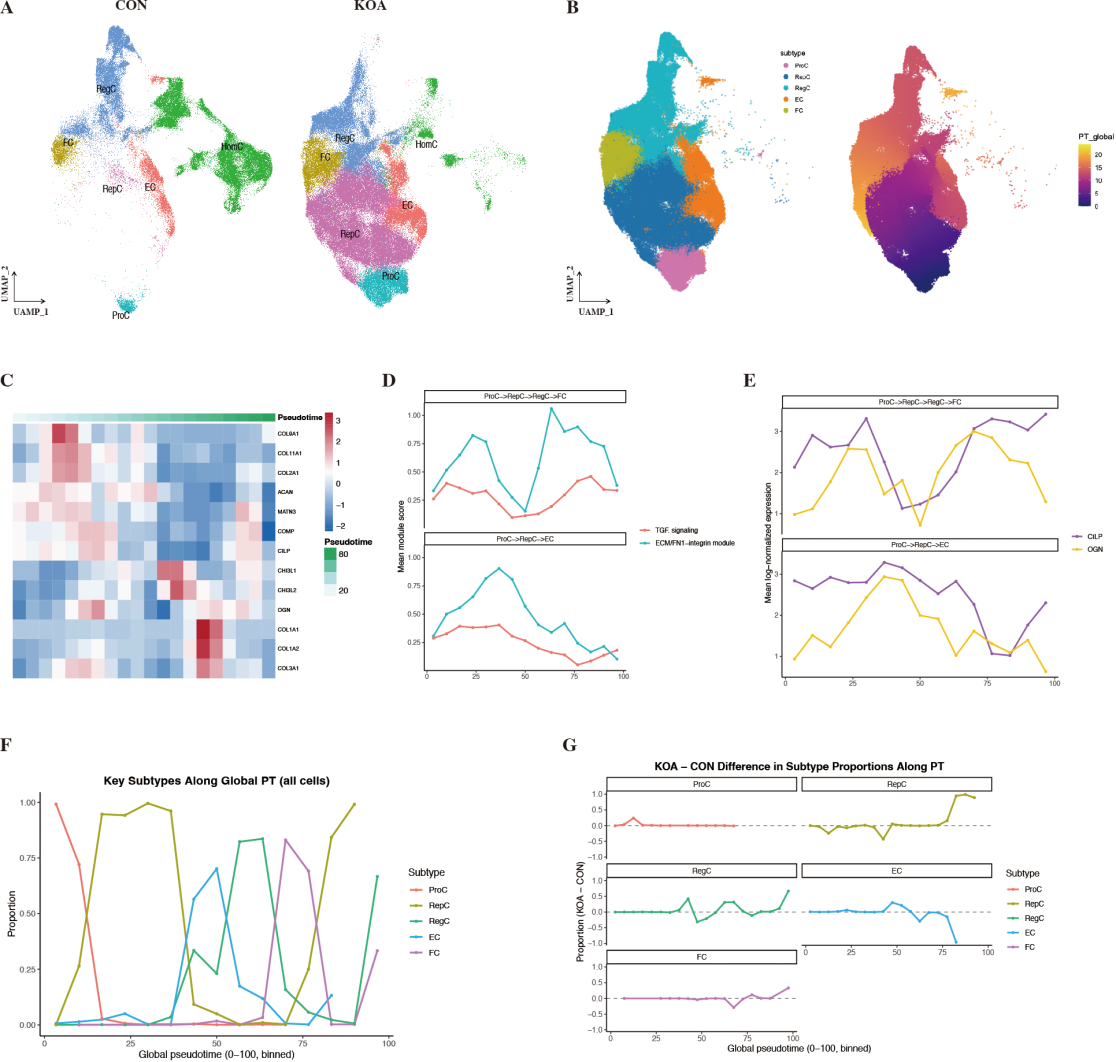
Pseudotemporal organization of chondrocyte states and RepC-centered branching structure. **(A)** UMAP visualization of chondrocytes colored by condition (CON vs. KOA) and annotated subtypes. **(B)** Global pseudotime ordering inferred by Slingshot, showing a continuous trajectory from ProC through RepC toward multiple downstream states. **(C)** Heatmap of dynamically expressed genes along global pseudotime, highlighting stage-specific expression patterns of ECM-related genes. **(D)** Module scores of ECM/FN1–integrin and TGF-related signaling pathways along branch-specific pseudotime trajectories, illustrating distinct temporal dynamics across divergent paths. **(E)** Expression dynamics of representative RepC-associated genes (CILP and OGN) along different pseudotime branches. **(F)** Proportional distribution of major chondrocyte subtypes along global pseudotime. **(G)** Differences in subtype proportions between KOA and control samples along pseudotime, showing enrichment of RepC and concomitant reduction of ECM-homeostatic states in KOA within the reconstructed trajectory framework.

Analysis of chondrocyte subpopulation distributions revealed that RepC exhibited a distinct and concentrated distribution pattern under KOA conditions compared with other subpopulations (**Figure 3A**), suggesting its association with a specific region of the reconstructed chondrocyte trajectory. To further define the relative positioning of RepC along continuous state transitions, pseudotime trajectory analysis was performed within the primary framework defined by ProC, RepC, RegC, FC, and EC. The inferred trajectory suggested an ordering from ProC toward RepC, after which the reconstructed framework bifurcated into two major branches: one extending toward RegC and FC, and the other toward EC (**Figure 3B**). Integration of gene expression dynamics and functional module scores along the pseudotime axis demonstrated that extracellular matrix remodeling–related programs were preferentially activated within the pseudotime interval corresponding to the RepC stage (**Figures 3C–E**). Quantitative assessment of subpopulation composition across pseudotime further showed that RepC was significantly enriched in the middle-to-late portion of the reconstructed trajectory (**Figures 3F, G**).

### 3.5 Cell–cell communication analysis suggests prominent ECM-related communication features of RepC in KOA

**Figure 4:**
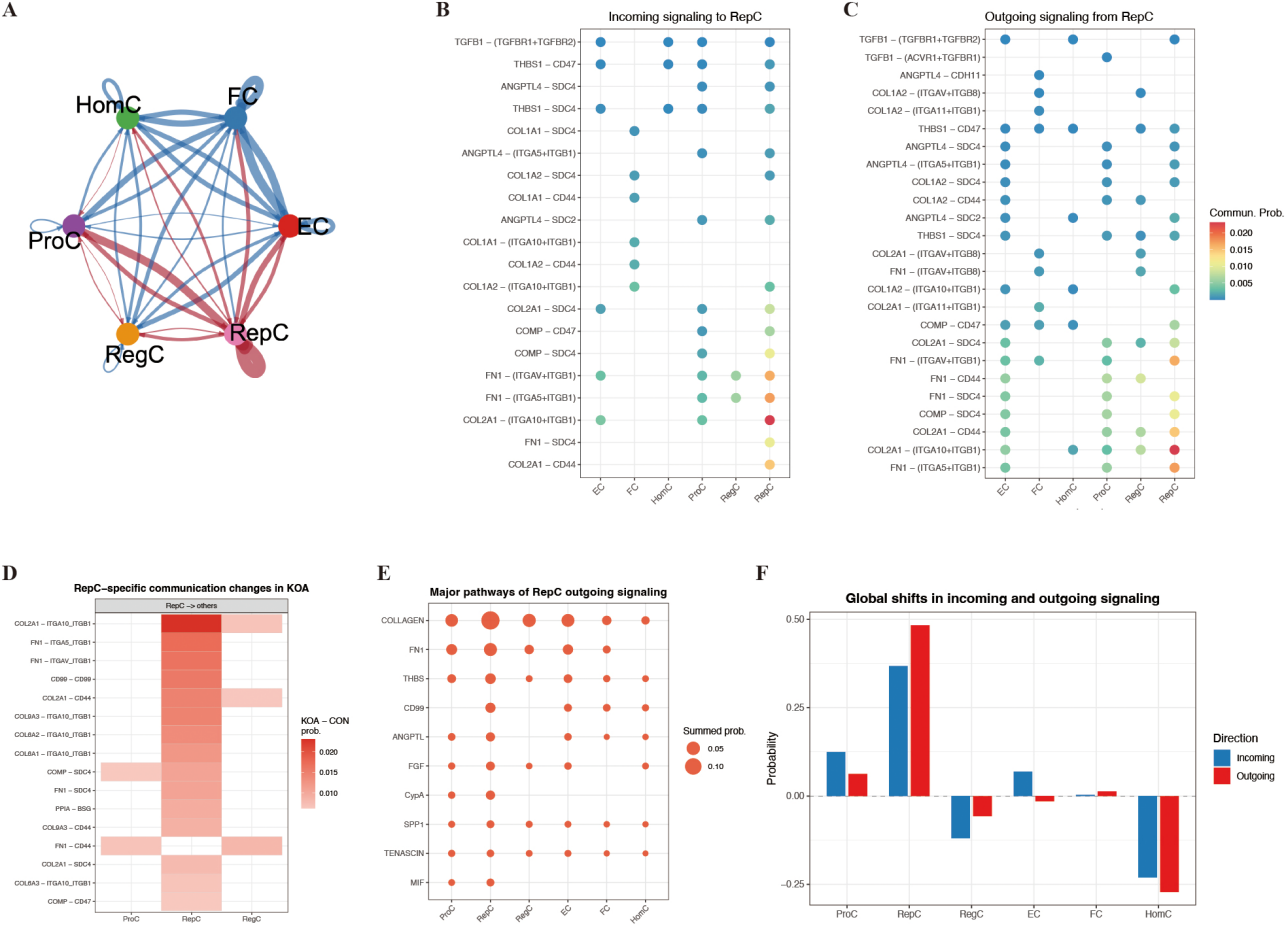
RepC-associated inferred cell–cell communication patterns among chondrocyte subtypes. **(A)** Global cell–cell communication network inferred by CellChat, highlighting RepC as showing relatively prominent inferred communication activity among chondrocyte subtypes. **(B, C)** Dot plots showing inferred incoming and outgoing communication patterns of RepC, dominated by collagen- and FN1-related ligand–integrin pairs. **(D)** Heatmap depicting RepC-associated changes in inferred communication probability between KOA and control conditions. **(E)** Summary of major inferred signaling pathways contributing to RepC outgoing communication, with enrichment of ECM-related pathways. **(F)** Global shifts in inferred incoming and outgoing communication probabilities across chondrocyte subtypes.

Having established the distinctive positioning of RepC within KOA-associated chondrocyte state transitions, we next examined its inferred role within intercellular communication networks (**Supplementary Figure 3**). Cell–cell communication analysis suggested that RepC exhibited relatively high inferred incoming and outgoing interaction probabilities within the global network and engaged in dense predicted interactions with multiple chondrocyte subpopulations (**Figure 4A**). At the level of inferred input, RepC was associated primarily with interactions involving COL2A1 and FN1; conversely, at the level of inferred output, RepC showed predominant interactions related to COL2A1 and FN1 with other subpopulations, indicating pronounced bidirectional communication patterns within the inferred network (**Figures 4B, C**). Comparative analysis between KOA and control conditions further suggested that RepC-associated COL2A1 and FN1 communication probabilities were significantly enhanced in KOA (**Figure 4D**). At the pathway level, this enhancement was reflected by an overall increase in signaling output dominated by COLLAGEN and FN1 pathways (**Figure 4E**). Under KOA conditions, both incoming and outgoing communication scores of RepC were elevated, whereas the corresponding communication scores in HomC exhibited a relative decline (**Figure 4F**).

### 3.6 TGF-β1– and FN1-associated signaling induces RepC molecular features *in vitro*

**Figure 5:**
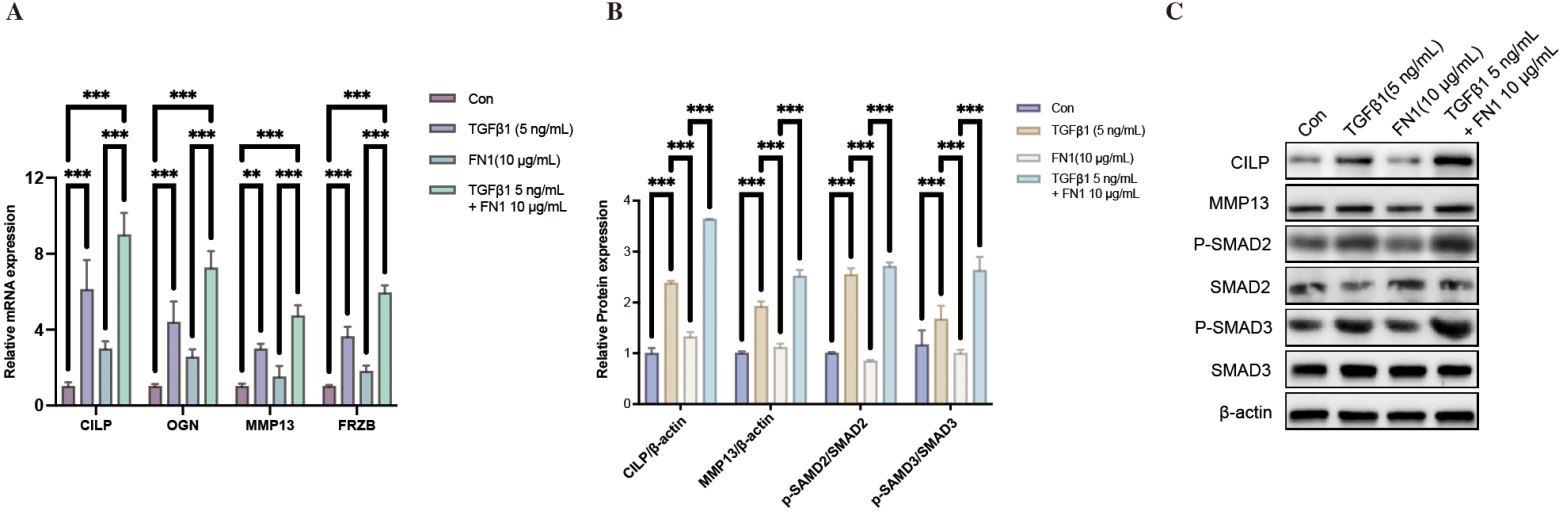
ECM-related cues promote RepC-associated gene expression and activate TGF-β/SMAD signaling in human articular chondrocytes. **(A)** Relative mRNA expression levels of RepC-associated genes (CILP, OGN, MMP13, and FRZB) in primary human articular chondrocytes treated with TGF-β1 (5 ng/mL), FN1 (10 μg/mL), or their combination. Gene expression was normalized to internal controls and expressed relative to untreated controls (Con). **(B)** Quantification of protein expression levels of CILP and MMP13, as well as phosphorylation levels of SMAD2 and SMAD3, under the same treatment conditions as in **(A)**. Protein levels were normalized to β-actin, and phosphorylation levels were normalized to total SMAD2 or SMAD3, respectively. **(C)** Representative immunoblot images showing CILP, MMP13, phosphorylated SMAD2 (P-SMAD2), total SMAD2, phosphorylated SMAD3 (P-SMAD3), total SMAD3, and β-actin under control, TGF-β1, FN1, and combined TGF-β1 + FN1 stimulation conditions. Data are presented as mean ± SD from n = 3 independent biological replicates. Statistical significance was determined by one-way ANOVA followed by Tukey’s *post hoc* multiple-comparison test. **p < 0.01, ***p < 0.001.

To assess whether RepC-associated molecular features identified in silico could be recapitulated under *in vitro* conditions, human articular chondrocytes were treated with TGF-β1 and FN1. Quantitative real-time PCR analysis (n = 3 independent biological replicates) demonstrated that, compared with control cells, treatment with either TGF-β1 or FN1 significantly increased the transcript levels of CILP, OGN, MMP13, and FRZB, whereas combined treatment further potentiated the induction of these genes (**Figure 5A**). At the protein level, Western blot analysis (n = 3 independent biological replicates) showed that TGF-β1 and FN1 treatment similarly enhanced the expression of CILP and MMP13 and was accompanied by increased phosphorylation of SMAD2 and SMAD3, with the most pronounced changes observed in the combined treatment group (**Figures 5B, C**).

## 4 Discussion

Osteoarthritis has traditionally been viewed as a disease driven by progressive cartilage degeneration and disruption of ECM homeostasis. However, chondrocytes are not merely passive and static participants in this process; rather, they display pronounced cellular heterogeneity and dynamic functional states (17, 18). With the advent of single-cell transcriptomic technologies, accumulating evidence has demonstrated that osteoarthritic cartilage comprises multiple chondrocyte subpopulations with distinct molecular signatures and functional biases, whose contributions to disease progression are not uniform (5, 8, 19). Previous studies have predominantly emphasized the pathogenic roles of inflammatory-like, hypertrophic-like, or fibrotic chondrocytes in osteoarthritis, while comparatively underappreciating the reparative responses activated by cartilage in the context of tissue injury (5, 20). Notably, cartilage tissue at early or intermediate stages of disease often exhibits substantial ECM remodeling activity. Such remodeling may reflect a compensatory response aimed at preserving tissue integrity, yet when dysregulated, it can promote fibrotic ECM remodeling and accelerate cartilage degeneration (21, 22). Accordingly, identifying and dissecting chondrocyte states that are closely linked to ECM remodeling is essential for understanding the shift from adaptive repair to pathological remodeling in osteoarthritis (18). Against this backdrop, although RepC has been reported in prior single-cell studies of osteoarthritic cartilage, the present study provides a more focused characterization of its enrichment pattern, trajectory positioning, and intercellular communication features within a unified multi-dataset analytical framework, with the aim of clarifying its disease-associated role in KOA rather than redefining it as a newly identified subtype.

Differential expression and functional enrichment analyses revealed that the alterations exhibited by RepC under KOA conditions were not merely quantitative but instead reflected a pronounced shift of its functional programs toward a highly active ECM remodeling state (19). In KOA, RepC was primarily characterized by the upregulation of programs associated with matrix reconstruction and extracellular matrix interactions, collectively defining a functional state dominated by collagen fibril reorganization and structural ECM remodeling. These findings suggest that RepC more likely represents an active response initiated by cartilage in the context of tissue injury, rather than a purely passive reaction to matrix breakdown (5). From a pathological standpoint, such ECM remodeling-associated reparative responses may be related to short-term attempts to preserve tissue integrity. However, when disproportionately represented or dysregulated, they may be associated with shifts in cartilage matrix composition and with a fibrocartilage-like remodeling pattern that accompanies structural deterioration during KOA progression (21, 22).

Based on cell annotation results, HomC were predominantly distributed in control cartilage, whereas their representation in KOA samples was limited. Because the aim of the pseudotime analysis was to resolve the major disease-associated continuum of chondrocyte state transitions in KOA, the trajectory shown here should be interpreted as a restricted framework rather than a complete map of all annotated chondrocyte subtypes. Accordingly, the primary pseudotime framework was constructed without inclusion of HomC. Within this framework, the remaining chondrocyte states displayed a continuous distribution within pseudotime space, with RepC positioned downstream of ProC and occupying a major branching region within the reconstructed trajectory framework toward multiple inferred trajectory branches. Pseudotime analysis indicated that upon entry into the RepC stage, cellular trajectories no longer progressed along a single linear path but instead diverged either toward RegC, with further extension to FC, or toward EC. These findings suggest that RepC is not merely a transitional intermediate, but rather a chondrocyte state occupying a distinctive position within the reconstructed KOA framework and associated with branch-specific transcriptional programs (23). Integration of global pseudotime analyses comparing subpopulation proportions between KOA and control conditions across different stages further demonstrated that KOA-associated alterations did not arise at the initial phase of the trajectory. ProC remained relatively stable between KOA and control groups, and changes at this stage were insufficient to explain the pronounced shifts observed in downstream state composition. Instead, the central abnormality in KOA was characterized by preferential representation of RepC at middle-to-late pseudotime stages. Concurrently, EC gradually declined within the same interval, suggesting relative underrepresentation of the branch associated with ECM-homeostatic features within the reconstructed trajectory framework (21). RegC exhibited moderate fluctuations during intermediate stages but increased markedly at later stages and, in certain branches, was accompanied by a further rise in FC, reflecting a progressive bias toward enhanced regulatory activity and fibrotic-related outcomes. Overall, this pseudotime-dependent redistribution of subpopulation proportions does not reflect aberrant expansion of a single cell type but instead reveals a RepC-centered imbalance in state transitions: a reparative phase that is relatively underrepresented under physiological conditions is disproportionately represented in KOA, with cells at this pseudotime stage showing a compositional bias toward regulatory and structural ECM remodeling-associated trajectories rather than homeostasis-associated outcomes (17).

Further interrogation of cell–cell communication revealed that RepC showed simultaneously elevated inferred incoming and outgoing communication probabilities within the chondrocyte communication network. Compared with other chondrocyte subpopulations, both incoming and outgoing inferred communication activities of RepC were coordinately increased, supporting a relatively prominent position of RepC within disease-associated communication remodeling. Under KOA conditions, this communication profile was largely organized around ECM-related signaling axes. At the level of signal input, RepC preferentially received extracellular matrix–associated ligand signals, including COL2A1 and FN1, originating from multiple chondrocyte states. Conversely, at the level of signal output, RepC predominantly transmitted signals to surrounding cells through FN1–integrin and COLLAGEN–integrin axes, forming a highly coherent bidirectional communication pattern (24–26). This inferred communication architecture, centered on ECM components and adhesion receptors while integrating regulatory cues such as TGF-β-related signaling, supports the view that RepC is not merely a passive responder to matrix alterations, but may occupy a relatively active position in ECM remodeling–associated communication. Nevertheless, because these interactions were inferred from ligand–receptor expression patterns, they should be interpreted as putative communication relationships rather than direct evidence of active signaling events (25). Consistent with this notion, comparative analyses revealed that the RepC-centered communication pattern was more prominent in KOA, with both incoming and outgoing inferred communication probabilities synchronously elevated at the network level, whereas other chondrocyte subpopulations did not display comparable changes. These observations suggest that KOA-associated communication remodeling is not globally enhanced but instead appears to be relatively concentrated in the repair-associated state represented by RepC. Such a shift is associated with preferential representation of RepC-centered ECM remodeling-related communication features within the KOA framework (27).

Intercellular interactions surrounding RepC were predominantly centered on matrix-related signals represented by collagen-associated ligands, with concurrent involvement of FN1-related adhesion signaling (28). However, cell–cell communication networks alone cannot determine whether such matrix-associated signals merely reflect intrinsic features of the RepC state or instead show trajectory-associated changes during inferred cell state ordering. To address this issue, we further interrogated these matrix-related signals within a pseudotime framework, focusing on their dynamic behavior at the RepC stage and along distinct branching trajectories. Our analyses revealed that matrix-related signaling was not uniformly distributed along the trajectory but was instead highly concentrated at the RepC stage. Along the global pseudotime axis, ECM/FN1–integrin–related modules were maintained at relatively low levels during the ProC stage and rapidly increased upon entry into RepC, indicating coordinated activation of functional programs associated with matrix reconstruction and cell–matrix adhesion at the RepC stage within the reconstructed trajectory. Subsequently, these modules displayed divergent patterns across different branches. Along the ProC→RepC→RegC→FC trajectory, the module peaked at the RepC stage and then partially declined, yet remained at relatively elevated levels during the middle-to-late pseudotime stages, suggesting that matrix-related programs were not completely terminated but instead underwent phase-specific reorganization and partial retention. In contrast, along the ProC→RepC→EC trajectory, the module rapidly decreased after RepC and approached baseline levels, indicating that matrix-related signaling was not preferential along this branch. In light of the preferential representation of RepC at middle-to-late pseudotime stages under KOA conditions, these differences suggest that functional programs associated with the trajectory branch leading away from RepC toward homeostasis-associated states are relatively underrepresented in the disease context. At the gene level, dynamic expression patterns of individual matrix-related molecules further underscored this branch-specific heterogeneity. Along the RegC→FC direction, CILP continued to increase following the RepC stage and remained highly expressed at later trajectory stages, whereas OGN gradually declined upon entry into later stages, indicating functional divergence among matrix-related genes after RepC rather than synchronized regulation. By contrast, along the branch leading toward EC, both genes exhibited only transient upregulation at the RepC stage followed by progressive attenuation (29, 30). Concurrently, collagen genes associated with hyaline cartilage homeostasis, including COL9A1 and COL11A1, were predominantly enriched during the early-to-middle pseudotime stages, whereas collagens linked to structural ECM remodeling, such as COL1A1, COL1A2, and COL3A1, gradually increased toward the later stages of the trajectory, reflecting a directional reorganization of matrix composition following RepC (20). Collectively, these findings delineate a RepC-centered organizational pattern in which the RepC pseudotime stage corresponds to coordinated activation of matrix–adhesion–related programs, and subsequent trajectory branches differ in the degree to which these programs are maintained or attenuated. This pattern is consistent with the observation that RepC is disproportionately represented in KOA and is associated with a compositional bias toward structural ECM remodeling–related outcomes.

Matrix-related programs at the RepC stage displayed pronounced state dependency, with substantial differences in activation across distinct branching trajectories, indicating that this functional state has a high capacity to respond to extrinsic signals. On the basis of this feature, we applied *in vitro* stimulation conditions in cultured human articular chondrocytes selected on the basis of features inferred from the single-cell analyses, in order to evaluate the molecular responsiveness of selected RepC-associated markers to matrix-related signals. These experiments were not designed to directly model state transitions or trajectory bias, but rather to assess whether key RepC-associated molecular features can be recapitulated under defined stimulation conditions. Our results demonstrated that treatment with either FN1 or TGF-β1 induced upregulation of multiple RepC-related genes, including CILP, OGN, MMP13, and FRZB, whereas combined treatment further amplified these changes, consistent with a synergistic effect (31). At the protein level, the increased expression of CILP and MMP13 was accompanied by enhanced phosphorylation of SMAD2/3, consistent with activation of signaling pathways associated with the RepC stage identified along the pseudotime trajectory. Together, these *in vitro* findings reinforce the dynamic features of extracellular matrix–related and cell adhesion–associated programs characteristic of the RepC stage in single-cell pseudotime analyses, supporting the interpretation that RepC-associated molecular features are responsive to matrix-related extrinsic cues and can be partially recapitulated under FN1- and TGF-β1-related stimulation conditions *in vitro*.

Several limitations of the present study warrant acknowledgment. First, the cell–cell communication analysis was performed using CellChat, which infers potential interaction probabilities from ligand–receptor expression patterns rather than directly measuring signaling activity. Therefore, ECM-dominant interactions involving genes such as COL2A1 and FN1 may reflect not only potential communication relationships but also the high expression of matrix-associated genes in specific chondrocyte states, and should be interpreted as inferred communication architecture rather than direct evidence of active signaling. Furthermore, network centrality metrics derived from CellChat reflect computational properties of the inferred communication network and should not be interpreted as direct evidence of biological dominance, integrin activation, or downstream signaling activity. Second, pseudotime analysis represents a computational ordering of cells based on transcriptional similarity and does not directly reflect true temporal progression; thus, trajectory-related interpretations should be understood as relative state ordering and compositional distribution within the reconstructed framework rather than as direct evidence of real-time cellular transitions. In addition, because the primary pseudotime framework was constructed without inclusion of HomC due to its markedly uneven distribution between conditions, the resulting trajectory model may be less informative for capturing potential return-to-homeostasis dynamics and should be interpreted with this limitation in mind. Finally, the *in vitro* experiments were performed using FN1- and TGF-β1-stimulated P2 human articular chondrocytes, a simplified system that does not fully recapitulate the mechanical loading, inflammatory milieu, and complex matrix microenvironment of KOA cartilage. These experiments therefore provide supportive molecular evidence consistent with RepC-associated features, rather than direct validation of disease-associated trajectory dynamics or inferred intercellular signaling.

## 5 Conclusion

By integrating single-cell transcriptomic analyses, pseudotime trajectory inference, cell–cell communication profiling, and *in vitro* functional assays, this study provides a systematic characterization of the ECM remodeling-related features, trajectory positioning, and intercellular communication patterns of RepC in KOA, building upon prior reports of this chondrocyte population in osteoarthritic cartilage. Our findings demonstrate that KOA-associated alterations do not primarily arise from progenitor-like stages but instead are characterized by preferential enrichment of RepC and a compositional bias in its downstream trajectory branches. Pseudotime analyses further indicate that RepC occupies a major branching region within the reconstructed trajectory framework, with extracellular matrix–related and cell adhesion–associated functional programs exhibiting selective activation and differential retention across distinct branching paths. Consistent with these single-cell observations, *in vitro* experiments reveal that RepC-associated molecular features are highly responsive to ECM-derived extrinsic cues. Collectively, these results suggest that RepC, previously recognized as a reparative chondrocyte population, shows preferential enrichment and ECM remodeling–related features in KOA and may represent a cell state associated with cartilage remodeling during disease progression.

## Data Availability

The original contributions presented in the study are included in the article/**Supplementary Material**. Further inquiries can be directed to the corresponding author.

## Supplementary material

Supplementary Figure 1 Quality control metrics and batch effect correction across integrated scRNA-seq datasets. **(A)** Quality control metrics for all samples prior to integration, including the number of detected genes per cell, UMI counts per cell, and mitochondrial gene percentages, shown separately for control (CON) and KOA samples across the three datasets. **(B)** Principal component analysis (PCA) of all cells before (upper panel) and after (lower panel) batch correction using the Harmony algorithm, colored by individual samples from different datasets. **(C)** Two-dimensional projections of cells colored by dataset identity before (left) and after (right) Harmony correction.

Supplementary Figure 2 Sample-level proportions of chondrocyte subtypes in control and KOA cartilage. Boxplots showing the sample-level proportions of six chondrocyte subtypes (EC, FC, HomC, ProC, RegC, and RepC) in control (CON) and knee osteoarthritis (KOA) samples. Each dot represents one sample, and the y-axis indicates the percentage of the corresponding subtype among total chondrocytes in that sample. Group comparisons were performed using the Wilcoxon rank-sum test. HomC was significantly reduced in KOA, whereas RepC was significantly increased; no significant differences were observed for EC, FC, ProC, or RegC. ns, not significant; ****, p < 0.0001.

Supplementary Figure 3 Global overview of ligand–receptor interactions among chondrocyte subtypes inferred by CellChat. Dot plot showing predicted ligand–receptor pairs between different chondrocyte subtypes inferred by CellChat. Dot color represents inferred communication probability and dot size indicates statistical significance as estimated by the CellChat framework. This figure provides a comprehensive reference of chondrocyte–chondrocyte communication patterns supporting the analyses presented in the main figures.

Supplementary Table 1 Cell counts per sample across integrated single-cell RNA sequencing datasets. A total of 193,409 high-quality cells were retained following quality control and doublet removal across three publicly available datasets. Each row represents one sample. CON, control; KOA, knee osteoarthritis.

Supplementary Table 2 Concordant differentially expressed genes in RepC identified by both cell-level and pseudobulk analyses. Genes listed were significantly upregulated in KOA RepC relative to CON RepC under both analytical approaches (adj. p < 0.05). FC_cell, log2 fold change from cell-level Wilcoxon analysis; padj_cell, Benjamini–Hochberg adjusted p value from cell-level analysis; FC_pb, log2 fold change from pseudobulk analysis; padj_pb, adjusted p value from pseudobulk analysis. KOA, knee osteoarthritis; CON, control.
