# Supplementary figures and images for: ECM remodeling features in reparative chondrocytes during knee osteoarthritis
Source: Front Endocrinol (Lausanne). 2026 Apr 13;17:1773139. doi: 10.3389/fendo.2026.1773139 (PMC13111078; doi:10.3389/fendo.2026.1773139)

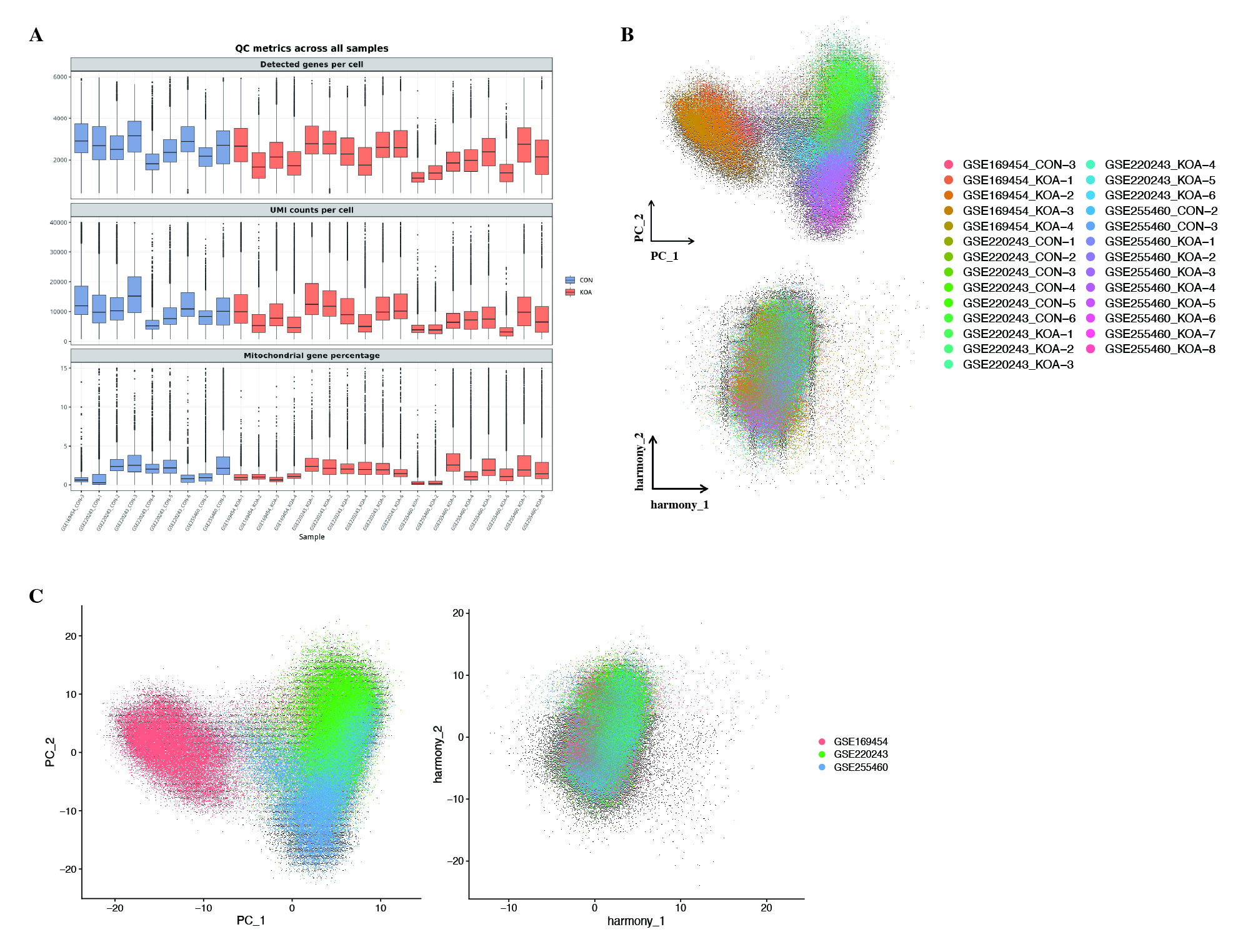

Supplement: Supplementary Figure 1 — Quality control metrics and batch effect correction across integrated scRNA-seq datasets. (A) Quality control metrics for all samples prior to integration, including the number of detected genes per cell, UMI counts per cell, and mitochondrial gene percentages, shown separately for control (CON) and KOA samples across the three datasets. (B) Principal component analysis (PCA) of all cells before (upper panel) and after (lower panel) batch correction using the Harmony algorithm, colored by individual samples from different datasets. (C) Two-dimensional projections of cells colored by dataset identity before (left) and after (right) Harmony correction. [file Image1.jpeg]

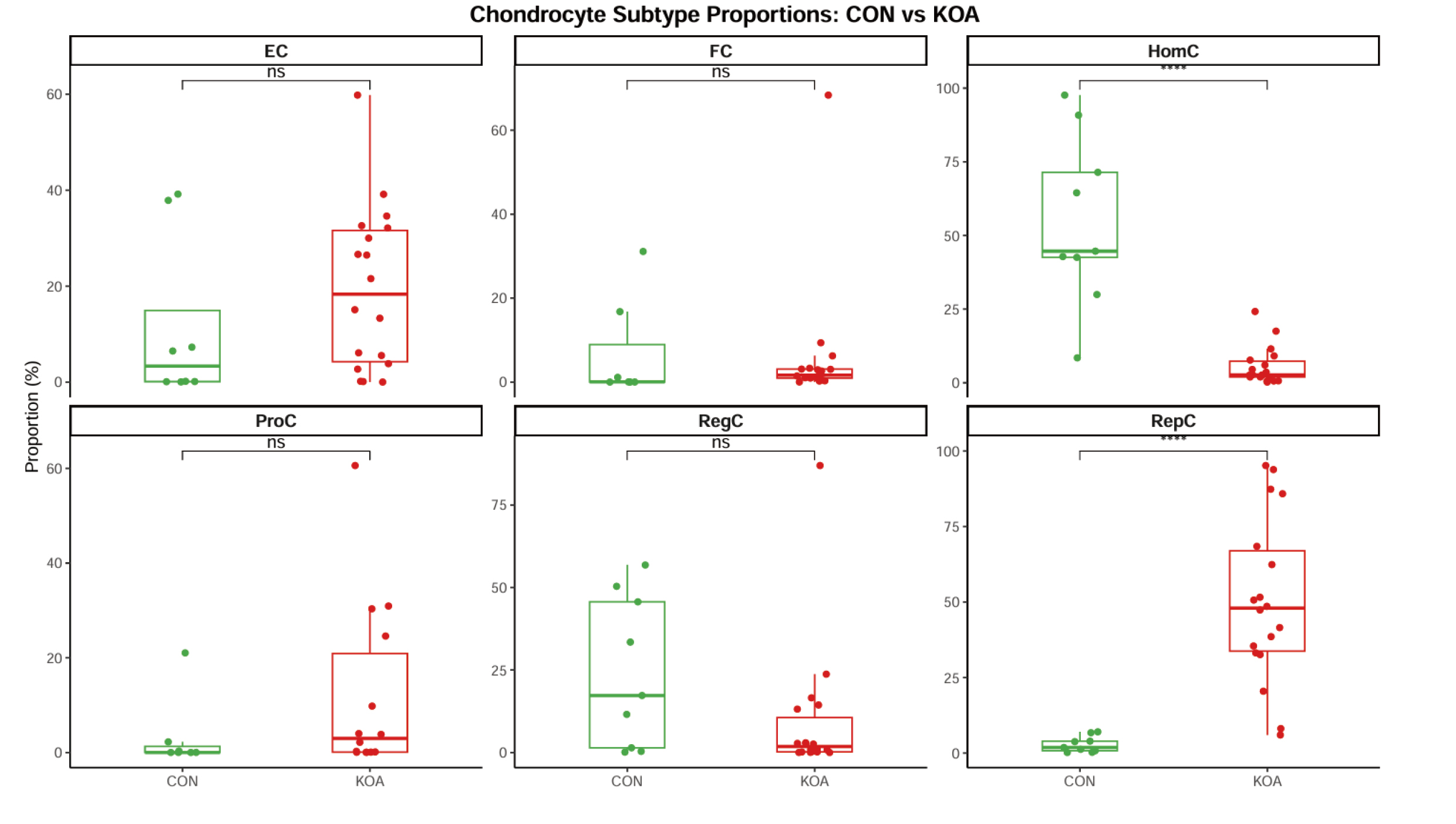

Supplement: Supplementary Figure 2 — Sample-level proportions of chondrocyte subtypes in control and KOA cartilage. Boxplots showing the sample-level proportions of six chondrocyte subtypes (EC, FC, HomC, ProC, RegC, and RepC) in control (CON) and knee osteoarthritis (KOA) samples. Each dot represents one sample, and the y-axis indicates the percentage of the corresponding subtype among total chondrocytes in that sample. Group comparisons were performed using the Wilcoxon rank-sum test. HomC was significantly reduced in KOA, whereas RepC was significantly increased; no significant differences were observed for EC, FC, ProC, or RegC. ns, not significant; ****, p < 0.0001. [file Image2.jpeg]

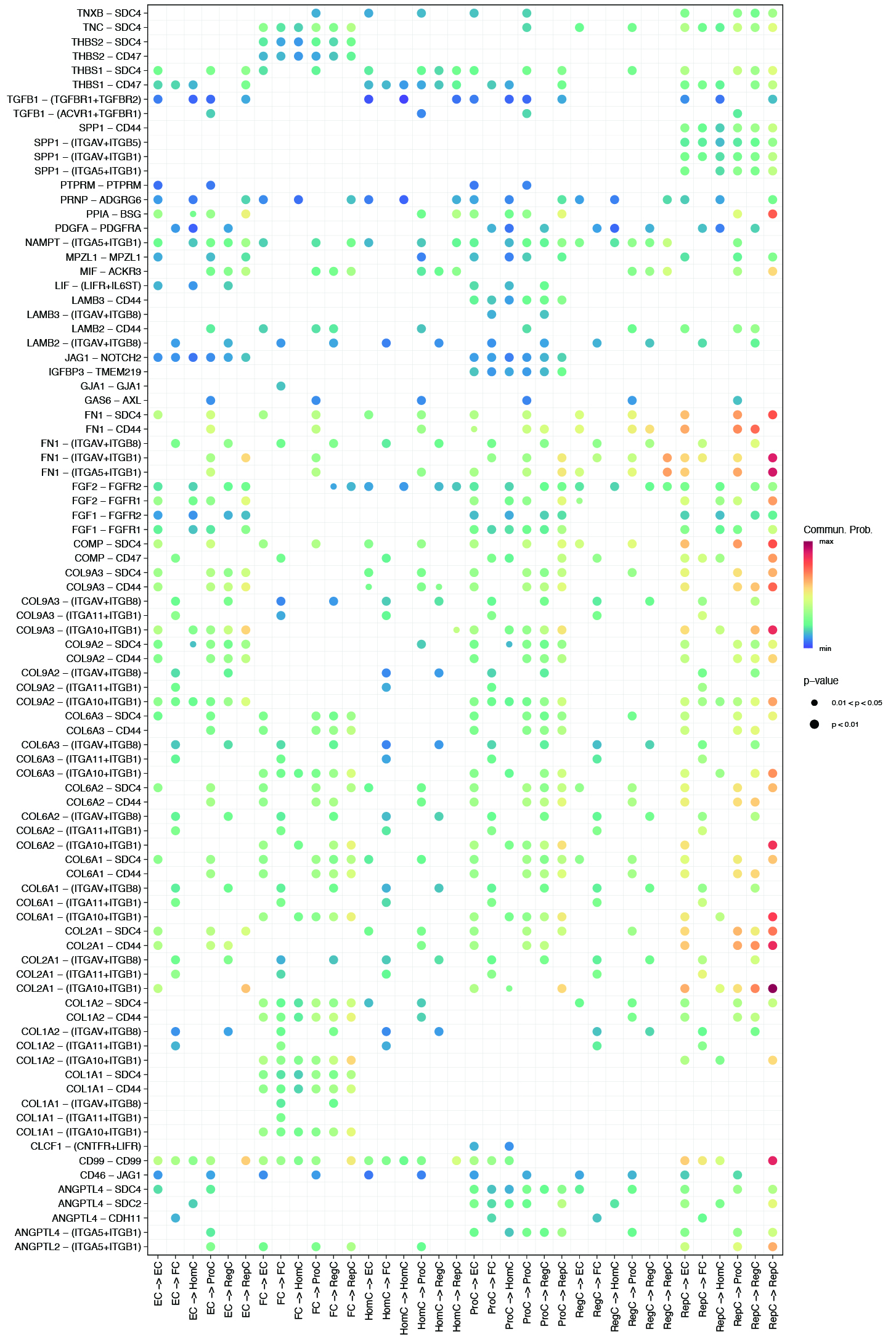

Supplement: Supplementary Figure 3 — Global overview of ligand–receptor interactions among chondrocyte subtypes inferred by CellChat. Dot plot showing predicted ligand–receptor pairs between different chondrocyte subtypes inferred by CellChat. Dot color represents inferred communication probability and dot size indicates statistical significance as estimated by the CellChat framework. This figure provides a comprehensive reference of chondrocyte–chondrocyte communication patterns supporting the analyses presented in the main figures. [file Image3.jpeg]
